# INfluence of Successful Periodontal Intervention in REnal Disease (INSPIRED): study protocol for a randomised controlled pilot clinical trial

**DOI:** 10.1186/s13063-017-2236-5

**Published:** 2017-11-13

**Authors:** Praveen Sharma, Paul Cockwell, Thomas Dietrich, Charles Ferro, Natalie Ives, Iain L. C. Chapple

**Affiliations:** 1 Periodontal Research Group, School of Dentistry, Institute of Clinical Sciences, Birmingham, B5 7EG UK; 2 College of Medical and Dental Sciences, University of Birmingham, and Birmingham Community Healthcare NHS Foundation Trust, Birmingham, B5 7EG UK; 3 Department of Renal Medicine, University Hospital Birmingham, Birmingham, B15 2GW UK; 4 Department of Oral Surgery, School of Dentistry, Institute of Clinical Sciences, Birmingham, B5 7EG UK; 5 Birmingham Clinical Trials Unit (BCTU), Institute of Applied Heath Research, Birmingham, B15 2TT UK

**Keywords:** Periodontitis, Chronic kidney disease, Randomised controlled trial, Periodontal treatment, Intervention, Pilot study

## Abstract

**Background:**

Patients with chronic kidney disease (CKD) exhibit increased morbidity and mortality which is associated with an increased systemic inflammatory burden. Identifying and managing comorbid diseases that contribute to this load may inform novel care pathways that could have a beneficial impact on the morbidity/mortality associated with CKD.

Periodontitis, a highly prevalent, chronic inflammatory disease affecting the supporting structures of teeth, is associated with an increased systemic inflammatory and oxidative stress burden and the successful treatment of periodontitis has been shown to reduce both.

This pilot study aims to gather data to inform a definitive study into the impact of successful periodontal treatment on the cardio-renal health of patients with CKD.

**Methods/design:**

This pilot study will employ a randomised, controlled, parallel-group design. Sixty adult patients, with CKD with a high risk of progression and with periodontitis, from the Queen Elizabeth Hospital, Birmingham, will be randomised to receive either immediate, intensive periodontal treatment (*n* = 30) or treatment at a delay of 12 months (*n* = 30). Patients will be excluded if they have reached end-stage renal disease or have received specialist periodontal treatment in the previous year. Periodontal treatment will be delivered under local anaesthetic, on an outpatient basis, over several visits by a qualified dental hygienist at the Birmingham Dental Hospital, UK. Patients in the delayed-treatment arm will continue to receive the standard community level of periodontal care for a period of 12 months followed by the intensive periodontal treatment. Randomization will occur using a centralised telephone randomisation service, following baseline assessments. The assessor of periodontal health will be blinded to the patients’ treatment allocation. Patients in either arm will be followed up at 3-monthly intervals for 18 months. Aside from the pilot outcomes to inform the practicalities of a larger trial later, data on cardio-renal function, periodontal health and patient-reported outcomes will be collected at each time point.

**Discussion:**

This pilot randomised controlled trial will investigate the viability of undertaking a larger-scale study investigating the effect of treating periodontitis and maintaining periodontal health on cardio-renal outcomes in patients with CKD.

**Trial registration:**

National Institute of Health Research (NIHR) Clinical Research Network (UKCRN ID: 18458), ID: ISRCTN10227738. Registered retrospectively to both registers on 23 April 2015.

## Background

Chronic kidney disease (CKD) affects over 13% of the adult population in the United Kingdom [1] and is associated with increasing age [2], hypertension and diabetes [3]. CKD is categorised into five stages, with stage 5 CKD, also known as established renal failure or end-stage renal disease (ESRD), comprising patients who may require renal replacement therapy (RRT) by dialysis or kidney transplantation. In 2009–2010, the annual cost for treatment of patients with stages 3–5 CKD in England was estimated at £1.45 billion, approximately 1.3% of the overall National Health Service (NHS) budget in that period; more than half of this was spent on patients requiring RRT [4].

The primary cause of mortality in patients with CKD is cardiovascular disease (CVD) [5]. Cardiovascular disease mortality in patients with CKD is not only related to the severity of kidney disease but also to an increased systemic inflammatory burden; biomarkers such as C-reactive protein (CRP) and interleukin-6 (IL-6) are reliable predictors of cardiovascular and all-cause mortality in patients with CKD [6, 7].

Consequently, identifying and targeting comorbid disease processes that contribute to systemic inflammation or oxidative stress burden, in patients with CKD, may lead to novel therapeutic approaches to reduce these burdens; an important strategy towards reducing mortality in such patients.

**Fig. 1:**
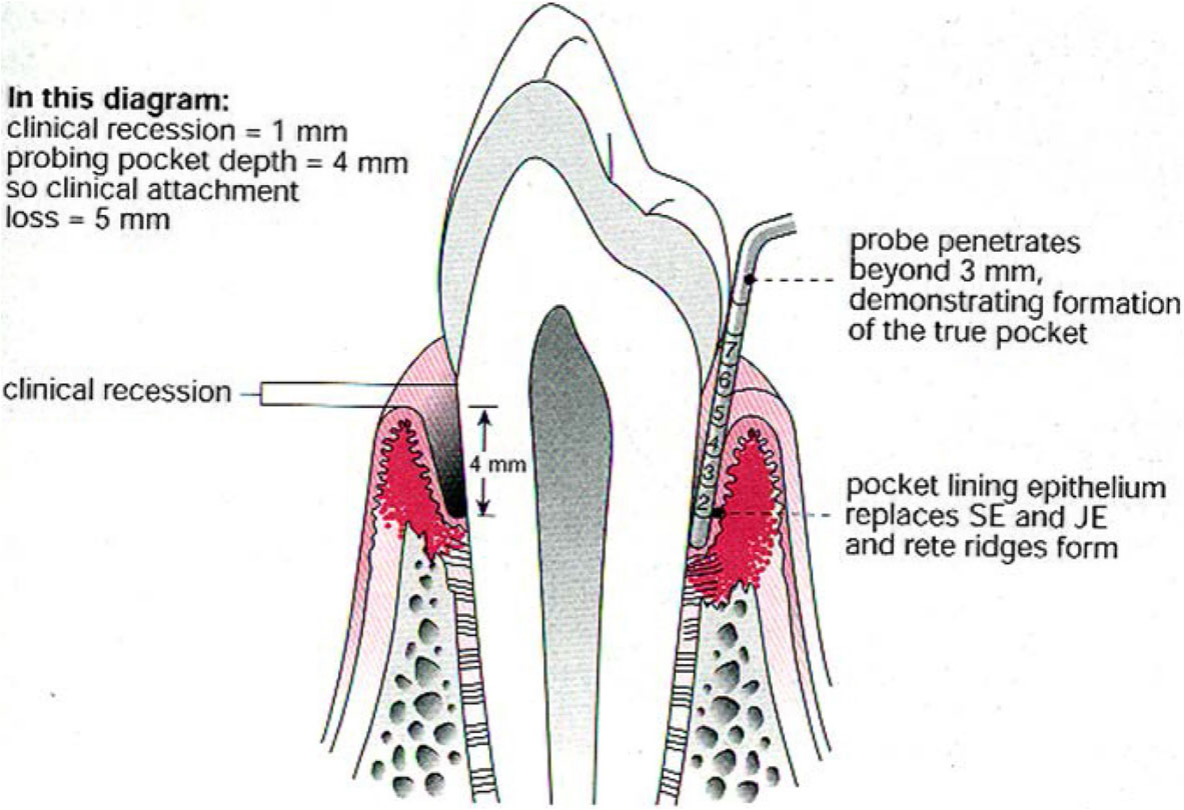
Anatomy of a tooth and supporting structures depicting destruction of the periodontal architecture due to periodontitis

Periodontitis is the most common chronic inflammatory condition in humans [8] and in its severe form is the sixth most common human disease, affecting 11.2% of the global population [9]. Periodontitis is initiated by bacterial accumulation between the gingivae (gums) and teeth, which triggers an inflammatory-immune response within the host. In susceptible individuals, the initial acute inflammatory response fails to resolve and a dysregulated chronic inflammation ensues, which destroys the supporting connective tissues surrounding the teeth. This results in periodontal ‘pockets’ forming, with chronically ulcerated pocket epithelium exposed to the microbial biofilm (Fig. 1 [10]). In severe disease the surface area of this ulcerated epithelium can be as large as 40 cm^2^ [11].

Periodontal inflammation contributes to the systemic inflammatory burden, through acute-phase and oxidative stress pathways [12], as evidenced by increases in CRP, IL-6 and biomarkers of oxidative stress in the serum of patients with periodontitis. Successful periodontal therapy is associated with reductions in these inflammatory mediators [13].

The association between periodontitis and other systemic diseases (particularly CVD) is well established [14, 15] and was recently reviewed in a joint European and American consensus workshop in periodontology [16].

Periodontitis may act as a comorbid chronic inflammatory disease in patients with CKD, contributing to increased systemic inflammation and the development of CVD. This risk pathway may be amenable to treatment as significant reductions in systemic inflammatory markers (IL-6, CRP) are reported following periodontal therapy in patients with CKD [17].

### Periodontitis and CKD

An ongoing longitudinal study investigating novel risk factors in the progression of CKD [18] has reported that patients with CKD at a high risk of progression, had a higher prevalence of periodontitis (odds ratio (OR) 4.0 95% CI 2.7–5.9) or severe periodontitis (OR 3.8 95% CI 2.5–5.6) compared to a local, control population [19].

We have recently analysed the US Third National Health and Nutrition Examination Survey (NHANES III, 1988–1994) database, for associations between periodontitis and mortality in patients with CKD and demonstrated a 10-year all-cause mortality of 41% (95% CI 36–47%) in patients with periodontitis compared with 32% (95% CI 29–35%) in patients without periodontitis [20].

To date, only a limited number of underpowered, non-randomised interventional studies have investigated the effect of periodontal therapy on renal function [21–23]. These studies have not answered the question of whether effective periodontal prevention and treatment may reduce both the morbidity associated with ESRD (dialysis or transplantation) and also the mortality associated with CKD.

## Research question

Our long-term, overarching goal is to evaluate whether treatment of periodontitis and periodontal maintenance can reduce renal and cardiovascular morbidity and mortality in patients with CKD.

As the research in this field is lacking, estimates of effect size are not available to adequately inform a sample size calculation. Furthermore, there are methodological considerations that need testing in a small-scale study, prior to embarking on a larger-scale, appropriately powered study. Therefore, the current pilot study was designed.

The specific research questions that this pilot study will address are:

1. Can 60 patients with CKD and periodontitis be recruited, screened and randomised into two treatment arms? What are the challenges in recruitment, screening or the randomisation process that need addressing?
2. Will patients find the intervention and follow-up appointments acceptable?
3. Are the proposed data collection methods acceptable?
4. Are patients willing to attend outpatient clinics for follow-up assessments and complete the trial assessments?
5. Does the data collected allow for the identification of a relevant and practical primary outcome measure for a larger study?
6. What are the barriers to clinical measurements and to collecting, storing and analysing samples?
7. What is an appropriate outcome measure/s to use in a subsequent, larger trial?
8. Are there changes needed in the study design/protocol and/or are there any barriers to a larger-scale study?

### Objectives and outcomes

#### Primary objective

As this is a pilot study, the primary objective is to inform a subsequent definitive trial. The pilot objectives will be achieved in answering the research questions detailed above. Selection of suitable primary and secondary outcomes, from the outcomes of interest listed below will inform subsequent sample size calculations for a pivotal trial.

The outcomes of interest include:

1. Measures of renal function (including estimated glomerular filtration rate (eGFR) and urinary albumin:creatinine ratio (ACR))
2. Measures of cardiovascular function (including blood pressure (BP), pulse wave velocity (PWV))
3. Measures of periodontal health
4. Patient-centred outcomes using the Oral Health Impact Profile-14 (OHIP-14) questionnaire

## Methods/design

The INSPIRED (INfluence of Successful Periodontal Intervention In REnal Disease) trial is a randomised, controlled, parallel-group pilot study and designed to address the research questions above. This trial was reviewed and favourably by West Midlands – The Black Country Research Ethics Committee (REC) (REC reference: 15/WM/0006) and is funded by a doctoral research fellowship grant by the National Institute of Health Research (NIHR), UK (grant reference: DRF-2014-07-109). The study is sponsored by the University of Birmingham (ref: RG_14-195). This manuscript is based on the latest version of the INSPIRED protocol (version 2.4, dated 28 Feb 2017) and is subject to change as the trial progresses. Any changes will be communicated to and authorised by the REC. The trial is registered online with the NIHR Clinical Research Network (UKCRN ID: 18458) and has the following ISRCTN identifier: ISRCTN10227738.

### Participants

**Fig. 2:**
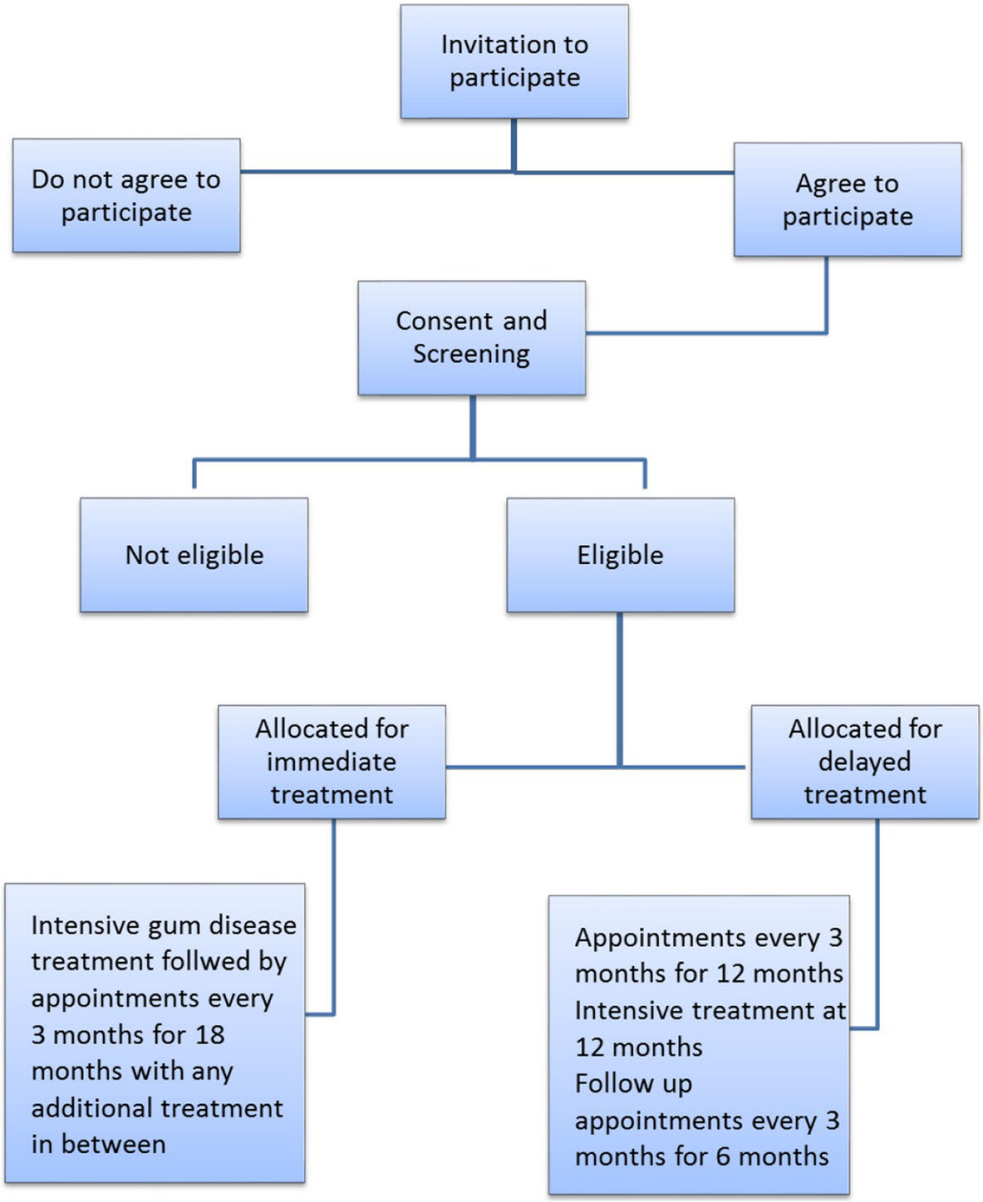
Flow of patients with chronic kidney disease (CKD) through the INSPIRED trial

**Fig. 3:**
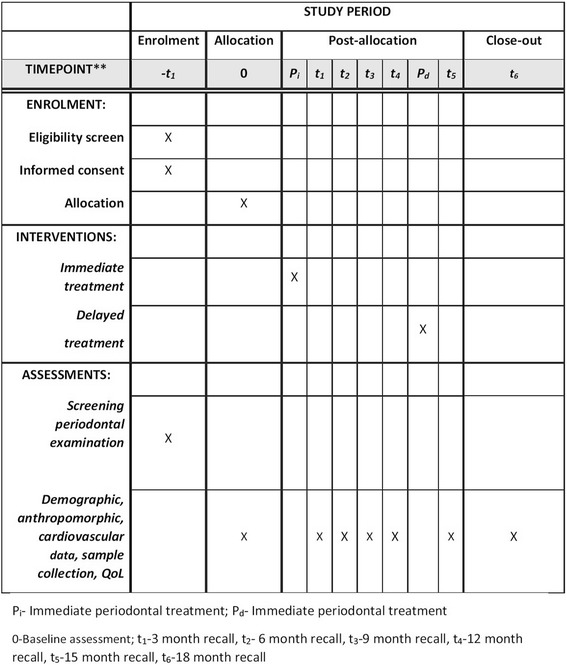
Standard Protocol Items: Recommendations for Interventional Trials (SPIRIT) Figure for the INSPIRED trial

Patients with CKD, with a greater likelihood of progression, as defined in the inclusion/exclusion criteria below, and periodontitis will be invited to participate in the INSPIRED trial. Participants for the INSPIRED trial will be recruited either from an existing, observational study in patients with CKD [24] or from patients with CKD attending clinics affiliated with the Queen Elizabeth Hospital, Birmingham, UK. The patient journey through the trial is illustrated in the flowchart (Fig. 2) as well as the Standard Protocol Items: Recommendations for Interventional Trials (SPIRIT) Figure (Fig. 3).

### Inclusion criteria

1. Patient aged 18 years or older
2. Able to provide informed consent to participate in the trial
3. Secondary care renal clinic follow-up for at least 1 year prior to recruitment
4. High-risk CKD defined as:
  (i) A decline of eGFR of 5 ml/min/year or 10 ml/min/5 years; and/or
  (ii) urinary ACR > 70 mg/mmol on three occasions; and/or
  (iii) CKD stage 4 or 5 (not on dialysis)
5. Generalised moderate-severe periodontitis defined as a minimum cumulative probing depth of 30 mm. This is the sum of the deepest probing pocket per tooth, excluding probing depths < 5 mm

### Exclusion criteria

1. ESRD requiring treatment with RRT
2. Receiving immunosuppression
3. Received specialist periodontal treatment in the previous 1 year
4. Not amenable to periodontal treatment, e.g. severe bleeding disorders that contraindicate periodontal treatment.

### Interventions

#### Immediate-treatment arm

This will consist of patients with CKD and periodontitis who are randomised to the immediate-treatment arm (30 patients). The patients will receive intensive periodontal treatment including oral hygiene instruction, supra and subgingival scaling and non-surgical root surface debridement followed by periodontal maintenance therapy. This will be provided by a qualified, experienced dental hygienist at the Birmingham Dental Hospital, UK. The root surface debridement will be performed on an outpatient basis under local anaesthesia over three or four visits (approximately 45 min each) approximately 1 week apart. Patients will be supported by a maintenance programme, for the duration of the trial, which will include a detailed periodontal examination to identify any recurrent disease early and facilitate any remedial treatment, if indicated.

#### Delayed-treatment arm

This will consist of patients with CKD and periodontitis who are randomised to the delayed-treatment arm (30 patients). Patients allocated to the delayed-treatment arm will still be eligible to receive the standard of care within the NHS, which is standard community level of periodontal care, as they would if they had not participated in the study [25]. Patients in this arm will have their oral and systemic health closely monitored at 3-monthly intervals for 12 months. After the 12-month review, patients in the control arm will be offered identical intensive periodontal treatment to those in the immediate-treatment arm. This will be done to facilitate recruitment and retention through assuring patients in the control arm that they will have future access to the same intensive treatment as patients in the intervention arm.

As chronic periodontitis is slowly progressing, usually over decades, there is no provision for modifying the allocation of patients from the delayed-treatment arm to the immediate-treatment arm

### Data and sample collection

Data will be collected on the periodontal status of all teeth present using a UNC-15 periodontal probe. The probing pocket depth (PPD) will be measured to the nearest millimetre from the base of the periodontal pocket to the gingival margin and recession will be measured to the nearest millimetre from the cement-enamel junction (CEJ) to the gingival margin (Fig. 1). For each tooth, PPD and recession will be measured at six sites – the mesial, mid and distal aspects of the buccal and palatal/lingual surfaces. The total clinical attachment loss (CAL) will be recorded as the sum of the probing depths and recession. Bleeding on probing (BOP) will be recorded dichotomously (present or absent) for each site probed. A marginal bleeding score will be calculated using bleeding from the gingival margin. A plaque score will be calculated dichotomously as the presence or absence of plaque on four surfaces of each tooth present.

Trial participants will have saliva, subgingival plaque and gingival crevicular fluid (GCF) samples collected and analysed. Collection of saliva and GCF samples will allow assessment of inflammatory and oxidative stress markers in the oral environment. Plaque samples will be collected to gauge changes in the periodontal microbiome over time and following periodontal therapy.

Collection of blood samples from trial participants will allow the assessment markers of renal health, glycated haemoglobin levels, and inflammatory and oxidative stress markers.

Renal health will be measured using the eGFR formula as calculated by the four-variable modification of diet in renal disease (MDRD) equation [26] with serum creatinine that is IDMS (isotope dilution mass spectrometry) traceable. We will also calculate eGFR using the CKD-Epidemiology Collaboration creatinine-cystatin C equation [27] as a sensitivity analysis. As local clinical laboratories are reporting based on MDRD GFR at the time of protocol development, approval and commencement we will use this equation for assessment of the CKD 4 threshold (eGFR < 30 ml/min/1.73 m^2^) and report any variances with the CKD-EPI equation.

For assessing the rate of decline of kidney function, each participant will have at least four eGFR results available during the follow-up period to allow an accurate assessment of the rate of change of eGFR with time in patients who have accelerated progression of kidney disease [28]. This approach has now been validated by a number of studies [29, 30]. However, we recognise that this approach may not have the sensitivity to detect decline in kidney function in patients who are sustaining a lower rate of decline of eGFR (e.g. < 2 ml/min/year) which may still have long-term clinical significance, in particular for patients with an eGFR < 30 ml/min. Urine samples will be collected to assess urinary markers of renal disease including ACR.

Cardiovascular health will be measured by measuring blood pressure, the carotid to femoral PWV, a surrogate marker of arterial stiffness [31], and surrogate markers of inflammation (serum CRP, IL-6) and oxidative stress.

Patient-reported outcomes will be collected by conducting an interview with patients before and after periodontal therapy to assess the patient’s perception of any benefit. The OHIP-14 questionnaire is a validated short version (14 questions) of the original OHIP-49 questionnaire and will be used to measure the patient perspective of oral health. The questionnaire has good reliability, validity and precision [32].

Other details of participants such as anthropomorphic (height, weight and body measurements), demographic and socioeconomic data will also be assessed.

The planned measurements and samples will allow assessment of their potential as primary or secondary outcome measures in a definitive trial as well as evaluating the collection and processing protocols of such data for a definitive study.

Measurements will take place at the Birmingham Dental Hospital, UK, at baseline and during follow-up appointments as detailed below, for participants in both arms of this trial and will be performed by qualified members of the research team.

### Follow-up measurements

Periodontal and cardiovascular measures and biological samples (blood, saliva, urine, subgingival plaque and GCF) will be taken at baseline, prior to initiation of periodontal treatment, and then repeated at 3, 6, 9, 12, 15 and 18 months. Patient questionnaires will also be administered at the same time points.

Review appointments will be timed to allow assessment of the response to periodontal treatment and also allow assessment of the need for reinforcement of oral hygiene instructions and any further periodontal treatment required, in the immediate-treatment arm. Treatment will be performed to a clinical endpoint of periodontal stability, defined as:

1. Ninety percent of patients will have fewer than five sites or fewer than two teeth with PPD ≥ 5 mm at the end of therapy that bleed on probing
2. In 90% of patients, plaque scores will be ≤ 20% at 6 months
3. In 90% of patients, bleeding from the pocket base will be ≤ 10% at 6 months

For patients in the control arm, review appointments will allow for careful monitoring of oral and systemic health. At the 12-month time point, patients in the control arm will be offered intensive periodontal treatment allowing for assessment of their treatment response and reinforcement of oral hygiene instructions at the 15- and 18-month review appointments. Therefore, measurements at the 15- and 18-month time points represent post-treatment follow-up measurements for both arms.

### Sample size

Due to a lack of previous research to indicate a reliable primary clinical endpoint, a ‘conventional’ sample size for a pilot study was chosen to obtain meaningful estimates of effect sizes for the various outcome measures. This sample size is also informed by the prevalence of periodontitis in an existing cohort of patients with high-risk CKD with the same recruitment criteria as employed in this trial [18].

### Randomisation

The randomisation uses permuted block-randomisation, with variable block size, stratified by CKD stage (stages 1–3 vs. stages 4–5) and smoking status (never vs. ever). The randomisation code will be held securely at the Birmingham Clinical Trials Unit (BCTU) at the University of Birmingham. After obtaining patients’ informed consent and completion of the baseline assessments participants can be randomised into the INSPIRED trial.

### Allocation concealment

Allocation concealment of the randomisation of participants in the INSPIRED trial to the immediate- or delayed-treatment arms will be ensured by using a centralised, telephone randomisation service provided by the BCTU. The random sequence will be generated by staff within BCTU and independently of the clinical trial staff. Research nurses involved in the trial will telephone the BCTU and will be informed of the treatment allocation of the patients.

### Blinding

The assessor of periodontal health within this study will be blinded to the treatment allocation of the participants as the periodontal care will be provided by an independent operator. Blinding of patients or operator (dental hygienist) is not possible within this interventional trial. Blinding of the assessor of general health will not be possible for logistic reasons. The measurements taken by the assessor of general health, such as BP, PWV, body measurements, etc., are objective and hence will not to be influenced by knowledge of treatment arm. The medical assessor being unblinded negates the need for unblinding in the duration of the trial.

### Anticipated compliance issues

The successful maintenance of periodontal health relies heavily upon patients improving and maintaining their oral hygiene. There can be compliance issues associated with attaining and maintaining an adequate level of oral hygiene and home care on the part of the patient. Patients in either arm, following periodontal intervention, will be supported with this through reinforcement of oral hygiene instructions during treatment and follow-up visits along with maintenance periodontal therapy being provided as required.

Compliance with meticulous oral hygiene will be assessed using plaque scores, a dichotomous measure of the presence or absence of supragingival plaque, and bleeding scores, a dichotomous measure of the presence or absence of bleeding on periodontal probing. These scores will be used in individualised biofeedback as patient motivation tools.

With regard to compliance in maintaining appointments, patients will routinely be sent letters and text messages reminding them of their appointments. This will be reinforced by telephone calls to patients. We anticipate a retention rate of over 90% based on other RCTs carried out in the Periodontal Department of the Birmingham Dental Hospital [32]. This rate of retention is also an indicator of good compliance within research participants [33].

### Statistical analysis plan

#### Recruitment and retention

Data on the patients approached to enter the study will be analysed descriptively in terms of number of patients approached, number eligible and number randomised. Reasons for non-entry into the trial will be assessed, particularly in relation to patient eligibility criteria and reasons for patient refusal. Data on patients who do not complete the trial (e.g. withdrawals and those lost to follow-up) will also be collected throughout the study to allow assessment of patient retention rates, and reasons for non-completion of the trial.

Dropouts will be analysed as the number and proportion of patients who did not complete the trial overall, and by trial arm. Reasons for non-completion will be analysed descriptively.

#### Outcome data

Outcome data collected will be summarised using summary statistics and an exploratory analysis will be performed using an intention-to-treat approach. Continuous variables will be summarised using means and standard deviations and categorical variables will be summarised using frequency tables. Appropriate graphical methods will be used in conjunction with these.

The differences between the arms in the means and mean change from baseline to each time point will be calculated, along with the 95% confidence intervals. For dichotomous variables, changes in proportions, instead of means, will be analysed over time. This will help to determine the sensitivity of the outcome measures, such as eGFR, ACR, PWV and measures of inflammation or oxidative stress to change following periodontal therapy.

#### Frequency of analyses

Analyses will be carried out using data from 3, 6, 9, 12, 15 and 18-month review appointments when patients retained in the trial have reached the 18-month review appointment. This will be done at the end of the trial and no formal interim analyses are planned as part of this pilot study.

#### Dissemination policy

The results from this pilot study will be disseminated via oral and poster presentations in national and international conferences in the dental and medical (renal) disciplines. If applicable, results will also be published as open-access publications in peer-reviewed journals in both the renal and dental communities.

If appropriate, the wider dissemination of these results might take the form of a website for patients and practitioners to access. The European Federation of Periodontology (EFP) website will be utilised, in keeping with the EFP Manifesto (http://www.efp.org/efp-manifesto/manifesto.html), alongside media releases to a reporter with longstanding interest in periodontal and systemic health at Bloomberg News Centre and also via media outputs from the British Society of Periodontology (BSP). The dissemination amongst the renal community will be sought in conjunction with the British Renal Society (BRS).

## Discussion

Patients with existing CKD are at an increased risk of progression and mortality, arising primarily from adverse cardiovascular events [5]. The elevated risk is associated with an increase in the systemic inflammatory and/or oxidative stress burden [7, 6], which may be elevated by periodontitis as the treatment of periodontitis has been shown to reduce these systemic markers of inflammation in patients with and without CKD [13, 17].

The present pilot study aims to assess the feasibility of undertaking a larger scale study investigating the effects of successful periodontal treatment and maintenance of periodontal health on cardio-renal function, and ultimately on survival of patients with CKD. If this proves to be beneficial, then periodontal health may be an important factor in the management of patients with CKD.

This study is underway and challenges in recruitment and retention have already informed the management of the trial. Initially, this study was designed to employ a combination of a ‘classical’ parallel group RCT design and a cohort multiple randomised controlled trial (cmRCT) design [34], with the aim of recruiting patients from an on-going longitudinal cohort study [18] as a pool of eligible patients and controls. However, a greater than anticipated number of medical events, unrelated to periodontal treatment have been occurring in the longitudinal cohort study, resulting in insufficient patient recruitment. In addition to sourcing patients who fit the inclusion criteria from different sites, we also needed to relax the inclusion criteria from a “periodontal health” point of view. Initially, our data suggested sufficient patients would have disease that was sufficiently severe to allow for a threshold of cumulative probing depth of 40mm. This was relaxed to a cumulative probing depth of 30mm to allow for more patients to be eligible. The disadvantage of lowering the threshold is that, if a dose-dependent relationship exists between extent and severity of periodontitis and systemic ill-health, the treatment of periodontitis in such patients is likely to produce a lower magnitude of improvement in outcome measures than patients with more severe periodontitis. A change was also made in the total number of participants in each arm of the study. Initially, our data indicated 50 patients per arm would be achievable. This was scaled back to 30 patients per arm in response to the lack of eligible patients in the longitudinal cohort study. This will still be sufficient to inform a power calculation for a larger, multi-centre trial if such a trial appears to required.

## Trial status

As of September 2017, this trial is recruiting participants.

## Abbreviations

ACR: Albumin:creatinine ratio
BCTU: Birmingham Clinical Trials Unit
BOP: Bleeding on probing
CAL: Clinical attachment loss
CEJ: Cemento-enamel junction
CKD: Chronic kidney disease
CONSORT: Consolidated Standards of Reporting Trials
CRP: C-reactive protein
CVD: Cardiovascular disease
eGFR: Estimated glomerular filtration rate
ESRD: End-stage renal disease
GCF: Gingival crevicular fluid
IDMS: Isotope dilution mass spectrometry
IL-6: Interleukin-6
INSPIRED: INfluence of Successful Periodontal Intervention on REnal Disease
NHS: National Health Service
OHIP: Oral Health Impact Profile
PPD: Probing pocket depth
RCT: Randomised controlled trial
REC: Research Ethics Committee
RRT: Renal replacement therapy
